# Estuarine gradients dictate spatiotemporal variations of microbiome networks in the Chesapeake Bay

**DOI:** 10.1186/s40793-021-00392-z

**Published:** 2021-11-27

**Authors:** Hualong Wang, Feng Chen, Chuanlun Zhang, Min Wang, Jinjun Kan

**Affiliations:** 1 College of Marine Life Sciences, and Frontiers Science Center for Deep Ocean Multispheres and Earth System, Ocean University of China, Qingdao, China; 2 Institute of Marine and Environmental Technology, University of Maryland Center for Environmental Science, Baltimore, MD USA; 3 Department of Ocean Science and Engineering, Southern University of Science and Technology, Shenzhen, People’s Republic of China; 4 Shenzhen Key Laboratory of Marine Archaea Geo-Omics, Southern University of Science and Technology, Shenzhen, People’s Republic of China; 5 Southern Marine Science and Engineering Guangdong Laboratory (Guangzhou), Guangzhou, China; 6 Microbiology Division, Stroud Water Research Center, Avondale, PA USA; 7 Academy for Advanced Interdisciplinary Studies, Southern University of Science and Technology, Shenzhen, People’s Republic of China

**Keywords:** Estuarine gradients, Planktonic microbiomes, Co-occurrence, Network stability, Chesapeake Bay

## Abstract

**Background:**

Annually reoccurring microbial populations with strong spatial and temporal variations have been identified in estuarine environments, especially in those with long residence time such as the Chesapeake Bay (CB). However, it is unclear how microbial taxa cooccurr and how the inter-taxa networks respond to the strong environmental gradients in the estuaries.

**Results:**

Here, we constructed co-occurrence networks on prokaryotic microbial communities in the CB, which included seasonal samples from seven spatial stations along the salinity gradients for three consecutive years. Our results showed that spatiotemporal variations of planktonic microbiomes promoted differentiations of the characteristics and stability of prokaryotic microbial networks in the CB estuary. Prokaryotic microbial networks exhibited a clear seasonal pattern where microbes were more closely connected during warm season compared to the associations during cold season. In addition, microbial networks were more stable in the lower Bay (ocean side) than those in the upper Bay (freshwater side). Multivariate regression tree (MRT) analysis and piecewise structural equation modeling (SEM) indicated that temperature, salinity and total suspended substances along with nutrient availability, particulate carbon and Chl *a*, affected the distribution and co-occurrence of microbial groups, such as Actinobacteria, Bacteroidetes, Cyanobacteria, Planctomycetes, Proteobacteria, and Verrucomicrobia. Interestingly, compared to the abundant groups (such as SAR11, Saprospiraceae and Actinomarinaceae), the rare taxa including OM60 (NOR5) clade (Gammaproteobacteria), Micrococcales (Actinobacteria), and NS11-12 marine group (Bacteroidetes) contributed greatly to the stability of microbial co-occurrence in the Bay. Modularity and cluster structures of microbial networks varied spatiotemporally, which provided valuable insights into the ‘small world’ (a group of more interconnected species), network stability, and habitat partitioning/preferences.

**Conclusion:**

Our results shed light on how estuarine gradients alter the spatiotemporal variations of prokaryotic microbial networks in the estuarine ecosystem, as well as their adaptability to environmental disturbances and co-occurrence network complexity and stability.

**Supplementary Information:**

The online version contains supplementary material available at 10.1186/s40793-021-00392-z.

## Background

Planktonic microbiomes comprise both free-living organisms and those attached to particles, which is typical and visible in water columns of estuaries. The planktonic microbiomes in estuaries are one of the most active microbial communities [1] and they contribute primarily to the most productive environments on the planet [2]. For example, estuaries have a high CO_2_ flux (~ 0.25 Pg C y^−1^) between water and air, which is largely supported by the process of microbial decomposition and carbon fixation [3]. These microbiomes drive the estuarine biogeochemical processes of the elements for life [4]. They are powering the cycles of nutrients (e.g., carbon, nitrogen, phosphorus, and sulfur), with important impact on the composition of greenhouse gases in the atmosphere, the formation of algal blooms, and the integrity of estuarine ecosystems [5–7]. In fact, microbiomes are the foundation for the estuarine food chains and food webs [8–10], and their composition/distribution are important in the balance and stability of the entire estuary ecosystem.

Estuaries harbor a tremendous diversity of microbes. Due to the strong temporal and spatial gradients and surrounding land uses, the composition and distribution of the estuarine microbiomes are largely affected by human activities and climate/environmental changes [11–13]. Many studies have shown that spatiotemporal environmental variations enrich certain microbial taxa to dominate in estuaries, including the Chesapeake Bay [14], Delaware Bay [15], Sacramento-San Joaquin River Delta [16], Pearl Estuary [17], and Columbia River estuary [18]. In response to strong physical, chemical and biological gradients, estuarine microbiomes exhibit pronounced fluctuations in production and biomass [19, 20], as well as community composition [11, 21, 22]. It has also been shown that different microbial groups respond differently to spatiotemporal variations [11, 23]. These earlier studies provide important insights into the impact of spatiotemporal variations and other disturbances (such as anthropogenic pressures) on estuarine microbial community structure and dynamics. More interestingly, the spatial and temporal succession patterns are repeatable and predictable on an annual base [24–28], suggesting the microbial population dynamics are closely interrelating to their ambient aquatic environments. Annual selection pressure driven by environmental forcing has, through the induction of recurrent patterns in resource availability, predator–prey dynamics and microbial interactions, allowed for the assemblage of largely stable and resilient microbial communities [28]. Annually reoccurring patterns indicate that different microbiomes indeed have distinct niches with limited redundancy, otherwise different combinations of microbiomes would appear under the same conditions and prediction can be difficult [26]. These annually reoccurring assemblages and structure of microbiomes suggest their potential ecosystem functions (e.g., autotrophic or heterotrophic microbial production) are specialized and annually repeatable as well [24].

Functional traits of microbiomes are products of multiple populations within communities rather than those of a single population [29], and therefore correlations and associations among microbial taxa are critical to maintain ecosystem integrity and microbiome functionality. Different species or populations interact with each other to form complicated networks through various types of interactions, such as predation, competition and mutualism [30, 31]. Theoretical studies showed that communities in which a large proportion of members connected through positive links (i.e. positive correlations) are deemed to be unstable; in such communities, members may respond in tandem to environmental fluctuations, resulting in positive feedback and co-oscillation [31]. In contrast, ecological networks with compartmentalization and presence of negative relations could increase the stability of networks under disturbances [31–33]. For instance, high proportion of negative links could better balance the asynchronous dynamics and therefore stabilize co-oscillation in communities and promote stability of networks [31]. Further, modeling studies show that increasing strength of a few key correlations within a food web can destabilize trophic cascades, such as the “gatekeepers” [34], as removal of influencers causes a network to fragmentation [35]. In general, microbial associations (networks) play critical roles in maintaining community states, ecological niches and function distribution in the context of the microbiome [36].

Co-occurrence networks can reveal information on associations within microbiomes and stability of whole communities [31, 36, 37]. It has been increasingly used to infer microbial potential interactions [30, 38] in soils [39, 40], oceans [41, 42], coastal waters [43], lakes [44, 45], rivers [46], and even in metabolic modeling [47] and genomic surveys [48]. These correlation-based networks show important details of community rules reflecting ecological processes such as cooperation and habitat partitioning, and could represent mathematical associations among different microbial groups [30, 37]. Despite co-occurrence networks have been studied in plenty of earlier studies, they rarely focused on the co-exist microbial networks and how these networks respond to spatiotemporal variations in estuarine environments such as the Chesapeake Bay (CB), the biggest estuary in North America with long residence time (up to 9 months) [49]. Taking into account the dynamic environmental gradients in CB such as temporal variations, freshwater runoffs and ocean water intrusion, microbial co-occurrence and their stability/resilience to environmental changes are critical in further understanding the CB ecosystem. As expected, not only the spatiotemporal variations have potentials to reorganize networks of associations between co-existing estuarine microbial taxa, the characteristics of these networks themselves can also determine the adaptability and resilience to environmental disturbances (such as agriculture and urban development). Nevertheless, there are still few detailed studies on microbial networks and their responses to environmental changes in typical estuarine environments [50–53], and we need to address this important knowledge gap to deepen our understanding of estuarine microbiome ecology.

Recently, we have characterized the microbial community structures across both temporal and spatial scales in the CB, where planktonic microbiomes were collected across seven sampling stations (along spatial and salinity gradients) in four seasons over three consecutive years [54]. In this study, we constructed co-occurrence networks based on the 16S rRNA gene high-throughput sequences. Significant and strong correlations (including both positive and negative) were included in co-occurrence network analysis [55]. Due to previously described predominant seasonal and spatial variations in prokaryotic microbiome structure, networks from different seasons (temporal) and locations (spatial) were also constructed. In order to test the stability of co-occurrence networks, we assessed responsiveness of network fragmentation to removal of significant nodes (i.e., with highest betweenness centrality). Further, primary environmental drivers for microbial associations in the estuary were tested with multivariate regression tree analysis (MRT, [56]). Lastly, pathways that may explain how environmental gradients contribute to shaping estuarine microbiomes and their networks were identified and quantified with piecewise structural equation model (SEM, [57]). Based on all these analyses, quantitative associations of estuarine microbiome structures and the corresponding environmental drivers were proposed. This study provides the first snapshot on CB microbiome networks, the stability and adaptability, as well as their quantitative responses to environmental gradients, which are critical in improving our knowledge and understanding of the ecology of estuarine microbiomes.

## Methods

### Sample collection and characterization

Detailed description of sampling and environmental measurements have been described in previous studies [14, 54]. Briefly, surface water samples were collected from the CB at seven stations along the middle axis in February/March, May/June, August, and October from 2003 to 2005 (Additional file 7: Fig. S1). Total 500 mL water samples (below 2 m) were taken at each station and filtered immediately through 0.2 µm Millipore polycarbonate filters (Millipore Corporation, Billerica, MA, USA). The filters were stored at – 20 °C prior to DNA (deoxyribonucleic acid) extraction. Water temperature and salinity were recorded on board with a Sea-Bird 911 CTD (conductivity temperature depth, Sea-Bird Electronics, Washington, USA). Based on water temperature [14, 58], the samples collected in the Bay were divided into four seasons: winter (February and March), spring (May and June), summer (August) and autumn (October). Salinity and temperature were measured on site during the sampling cruises, but other abiotic data were obtained from the Chesapeake Bay Program's (CBP) Water Quality Database (https://www.chesapeakebay.net/what/downloads/cbp_water_quality_database_1984_present). These parameters were sampled/measured in the same month and close to the sampling days, including total organic nitrogen (TON), ammonium, nitrate, Chl *α* (Chlorophyll *a*), dissolved organic phosphorus (DOP), orthophosphate phosphorus (OP/phosphate), total suspended solids (TSS), particulate carbon (PC), and turbidity (measured as Secchi depth). Although the CBP stations do not have the exact same coordinates as our stations, they are reasonably close to our sampling sites (within 2–3 km).

### DNA extraction and sequencing analysis

Environmental DNA extraction followed the protocol described previously [59]. Dried environmental DNA pellets were lyophilized and archived in -80ºC for long time storage. The V4 region of the 16S rRNA (ribosomal ribonucleic acid) genes were amplified using the primers 515f (5′-GTGYCAGCMGCCGCGGTAA-3′) and 806r (5′-GGACTACNVGGGTWTCTAAT-3'), and sequenced on an Illumina Nova6000 platform (paired-end 250-bp mode) following the manufacturer’s guidelines. High-throughput sequences were processed and analyzed with QIIME (Quantitative Insights Into Microbial Ecology) 2_2019.1 [60]. DNA concentration and quality, sequencing depth, quality of raw reads and further results all demonstrated that the samples are qualified for sequencing analysis. Taxonomy annotation of these qualified sequences were based on 99% similarity to references in the Silva classifier 132-99-515-806 databases. The sequenced dataset contained 15,439,352 sequences constituting 4,865 amplicon sequence variants (ASVs) after removing the chloroplast and unidentified reads. These ASVs were rarified to 85,000 sequences per sample for downstream analyses. To avoid potentially erroneous sequences and improve interpretability of the dataset, we filtered out the ASVs (1) presented in fewer than 20% samples, and (2) with summed relative abundance less than 0.5% in each particular network inference [46]. After this sequence processing, the final dataset consisted of 6,002,328 sequences which constituted 780 ASVs for further co-occurrence network analysis. Raw sequence data is available at the GenBank database under the accession number SRX6973110-SRX6973185.

### Co-occurrence network analysis

Three categories of co-occurrence networks were constructed: (1) networks across all samples to explore overall microbial associations at spatiotemporal scales; (2) networks in four different seasons based on water temperature; and (3) networks in upper and lower Bay. Salinities were significantly different (*P* < 0.01) between the upper Bay (samples from station 908, 845, and 818) and lower Bay (samples from station 707, 724, and 744). In order to compare the networks across season and space, they were nomalized at 18 samples and 340 ASVs for seasonal networks and 31 samples and 384 ASVs for upper and lower Bay networks respectively, by using the function “sample” in R before network construction. Network enhancement was implemetated by the package ‘neten’, and applied to remove the fake linkes [61]. Microbial networks were constructed at ASV level. All network constructions were performed in R (version 4.1.0) and corresponding codes were adapted from GitHub (https://github.com/ryanjw/co-occurrence) [55]. The false discovery rate was estimated and corrected by the package ‘fdrtool’. Only microbial families with statistically significant (*P* < 0.01, Q-value < 0.05) and robust (spearman's correlation coefficient >|0.6| before and after network enhancement analysis) correlations [40] were included to present both of positive and negative relations in network analyses. In order to test if networks were significantly clustered, random networks were also constructed and compared following RJ Williams, A Howe and KS Hofmockel [55].

Network visualization and topological analysis were carried out in Gephi (version 0.9.2). The nodes with high degree or high betweenness centrality are crucial for ecological network structure and persistence because they literally hold the network together [62–65]. The topological properties of microbial networks were calculated with indexes including component, average clustering coefficient, modularity, network diameter, graph density, average path length, proportions of positive and negative correlations, and network fragmentation (*f*) [66–69]. Because microbial groups contribute differently to the network structures, the distribution of node-normalized degree (the number of connections a node has standardized by the total number of connections in the network) in each network was also examined. Microbial networks were also analyzed at the family level in order to mine universal microbial patterns.

Network stability were primarily characterized based on network topology such as number of components, modularity, proportion of negative correlations, and network fragmentation (*f*) (Additional file 1: Table S1). The *f* was calculated as the ratio of the number of disconnected subgraphs (CL) to the overall number of nodes (*N*) in each network as log(CL)/log(*N*) [46]. The *f* ranges from 0 to 1, and closer to 1 represents more fragmented and less stable networks. Loss of “gatekeepers” (*i.e.*, nodes with high betweenness centrality) contributes disproportionately to network fragmentation, suggesting high fragility of these networks upon selective removal of species [46, 70]. Therefore, the network stability was further tested by recording *f* upon iteratively removing the top 10 nodes with the most abundance, betweenness centrality, and degree [46, 71]. In addition, the correlation between node-normalized degree and betweenness centrality were explored: higher correlations indicated more stable networks because when a network containing more nodes with both high betweenness centrality and high degrees, it is more likely to have alternative nodes to hold the network together when certain nodes with high betweenness centrality were lost [36]. Based on all these network parameters, the stability of microbial co-occurrence was evaluated and compared across time and space.

### Multivariate regression tree (MRT) analysis

To determine how the CB microbiomes respond to spatiotemporal variations, multivariate regression tree analysis (MRT, [72]) was used to evaluate the hierarchical effect of environmental changes on the microbiomes. It was determined and generated by the R package “mvpart” (R version 3.6.1), and the divisions in the MRT were determined by cross-validation.

### Structural equation modeling (SEM)

Piecewise structural equation model (SEM) was used to analyze hypothetical pathways that may explain how spatiotemporal variations in the Bay shaped the composition, distribution and associations of microbiomes [73]. Compared to redundancy analysis that has been applied in our earlier papers [54], it allowed us to partition direct and indirect effects of each environmental variable relative to major bacterioplankton groups and estimate the strength of multiple effects. The first step was to build a *priori* model based on the known effects and relationships among environmental factors and dynamics of prokaryotic microbial communities. From MRT analysis results, we identified temperature, salinity and TSS as the primary driving factors. These three environmental variables covered seasonal variations and influence from freshwater or ocean input, and therefore affected microbial associations through changing Chl *α*, nutrient availability (*e.g.* N, P), and PC. Then, a maximum likelihood goodness-of-fit test was used in model fitting and a non-significant *P* value indicates a well-fit model [74]. In comparison to traditional SEM, piecewise SEMs are less restricted by the number of links per sample size and Fisher’s C can be used as the goodness-of-fit indicator [57, 75]. Piecewise SEM was performed in R (version 3.6.1) by using the piecewiseSEM package [57].

## Results

### Microbial networks across all samples

**Fig. 1:**
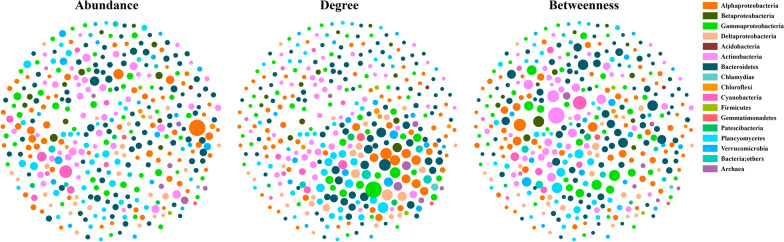
Co-occurrence networks of planktonic microbiomes in the Chesapeake Bay. Color coded nodes represent major bacterial ASVs. The relative abundance, degree or betweenness centrality of each ASV are shown by node sizes. Red lines and black lines represent significant negative or positive correlations

The correlation-based co-occurrence networks including all samples were constructed and visualized based on relative abundance, degree, and betweenness centrality, respectively (Fig. 1). The resulting CB microbiome networks consisted of 387 nodes (prokaryotic microbial taxa) and 4130 edges (significant and robust associations between taxa; average degree 21.3) (Fig. 1). Topological properties commonly used in network analysis were calculated and listed in Table 1. The average network path length between all pairs of nodes was 3.12 edges with a diameter (longest distance) of 8 edges. In total, the network contained 3 components (those separated subgraphs in a network) and 7 clusters (a group of nodes with higher number of within-cluster edges than between-cluster edges). The clustering coefficient (the degree that nodes tend to cluster together) was 0.509. These clusters were affiliated to almost all major prokaryotic microbial groups in the Bay, and also showed both positive and negative relations with many nodes in other clusters. Plentiful prokaryotic microbial groups clustered into one cluster with both positive and negative relations means that microbial associations were diverse and complex in the Bay. These clusters revealed the potential ecological relationships among prokaryotic microbiomes in the Bay and how they organized by niches in the estuarine ecosystem with habitat partitioning/sharing. Overall, the microbiome network in CB was comprised of highly interconnected taxa, formed a clustered topology, and contained ‘small-world’ properties (nodes are more connected) based on the modularity statistical analysis in Gephi.

**Table 1:** Topological properties of microbial co-occurrence networks in the Chesapeake Bay

Groups	Nodes	Edges	Fragmentation (*f*)	Average degree	Network diameter	Graph density	Modularity	Components	Average clustering coefficient	Average path length	Number of Clusters	Number of negative correlations	Percentage of negative correlations (%)
All	387	4130	0.18	21.3	8	0.055	0.513	3	0.509	3.118	**7**	**286**	**6.92**
All^a^	377	3917	0.23	20.8	8	0.055	0.506	4	0.506	3.137	**9**	**265**	**6.77**
All^b^	377	3525	0.19	18.7	8	0.050	0.54	3	0.497	3.163	**8**	**272**	**7.72**
All^c^	377	3855	0.27	20.5	8	0.054	0.498	5	0.509	3.222	**9**	**241**	**6.25**
Winter	331	3430	0.12	20.7	8	0.063	0.554	2	0.392	2.822	**9**	**526**	**15.34**
Spring	340	3296	0	19.4	8	0.057	0.576	1	0.413	2.866	**6**	**513**	**15.56**
Summer	338	3983	0	23.6	6	0.070	0.576	1	0.427	2.638	**6**	**720**	**18.08**
Autumn	337	3790	0	22.5	6	0.067	0.591	1	0.446	2.700	**6**	**707**	**18.65**
Upper Bay	367	4526	0	24.7	7	0.067	0.538	1	0.474	2.859	**7**	**344**	**7.60**
Lower Bay	376	5012	0	26.7	8	0.071	0.534	1	0.479	2.799	**7**	**559**	**11.15**

^a^After removal of top 10 nodes with the highest abundance

^b^After removal of top 10 nodes with the most degree

^c^After removal of top 10 nodes with the most betweenness centrality

Top ten ASVs ranked with averaged abundance, degree (number of connections) and betweenness centrality (how well a node is interacting simultaneously with different compartments of the network, potentially “gatekeepers”) were selected and listed in Additional file 2: Table S2. The high-degree nodes (*i.e.*, hub species that have the highest degree [36]) belonged to Gammaproteobacteria (Alcanivorax and SAR86 clade), Bacteroidetes (Rhodothermaceae and Ignavibacteria), Alphaproteobacteria (AEGEAN-169 marine group, SAR116 clade and Sneathiellaceae), Deltaproteobacteria (OM27 clade), Planctomycetes (Pla3 lineage), and Betaproteobacteria (Nitrosomonadaceae) (Additional file 2: Table S2). The nodes with high betweenness centrality belonged to Actinobacteria (Microbacteriaceae and Sporichthyaceae), Cyanobacteria (*Cyanobium* PCC-6307), Alphaproteobacteria (Pseudorhodobacter), Gammaproteobacteria (Ectothiorhodospiraceae and OM60(NOR5) clade), Betaproteobacteria (Polynucleobacter), Planctomycetes (*Pirellula*), Bacteroidetes (NS11-12 marine group and Fluviicola) (Additional file 2: Table S2). These nodes were crucial for microbial network structure and persistence in CB because they literally hold the network together. In contrast, the most abundant bacterial ASVs (*e.g.*, SAR11 clade, Saprospiraceae and Actinomarinaceae) are distinct from hub species and those groups with high betweenness centrality (Additional file 2: Table S2). Similar patterns were also observed at the family level (Additional file 8: Fig. S2).

### Microbial co-occurrence in different seasons

**Fig. 2:**
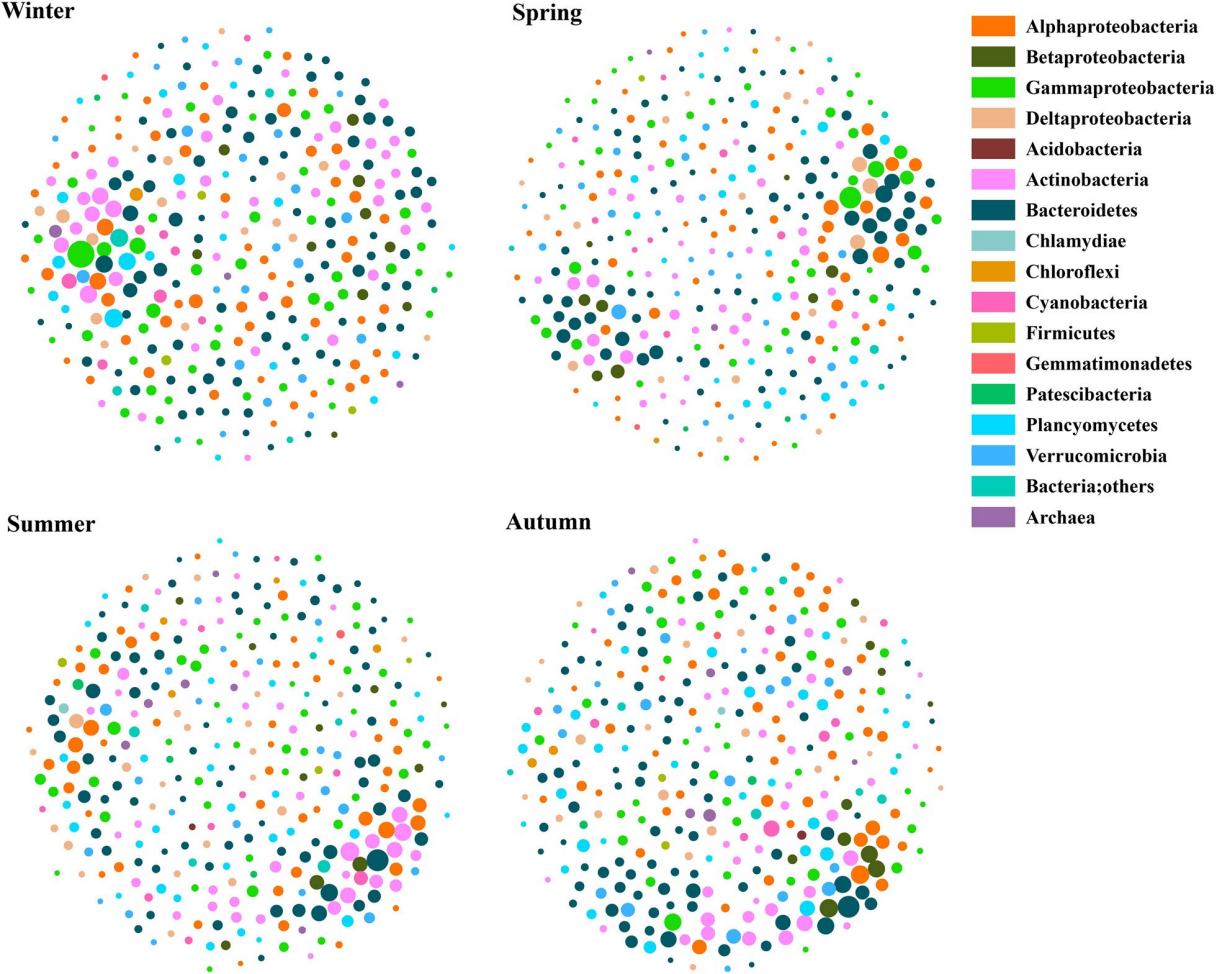
The architecture of microbial networks in winter, spring, summer, and autumn. Color-coded nodes represent the major bacterial ASVs within each network. Node sizes indicate number of connections (degree) for each node (ASV). Negative and positive correlations are shown in red or black lines

Distinct seasonal networks were obtained after applying the identical thresholds as described above (Fig. 2 and Table 1). For clarity and brevity, only networks based on microbial connections (degree) were included (Fig. 2). The seasonal networks were comprised of highly connected microbial taxa and densely connected groups formed a clustered topology with comparable seasonal variations (Fig. 2). The modularity indexes across four seasons were all greater than 0.4 suggesting modular structures especially in the autumn (Table 1) [76]. Within a co-occurrence network, modules are densely correlated microbial groups governed by ecological processes such as conserved inter-species communications, and therefore are good indicators for habitat partitioning/preferences [67]. The number of components in the network of each season was 1 except it’s 2 in the winter, and the amount of clusters was the same across seasons (6), except the winter network was 9 (Table 1). The summer network consisted of the biggest number of edges (3, 983) with average degree 23.6 (Table 1). Although the network in winter contained the lowest number of nodes (331), it had the high average degree (20.7) with 3, 430 edges compared to spring network (Table 1). In addition, the network in summer contained the highest graph density, shortest path length and high clustering coefficient (Table 1). All these characteristics indicated that summer microbiomes in CB were more closely associated and correlated.

The nodes with high degree/betweenness centrality were different for each season (Additional file 3: Table S3). For example, Betaproteobacteria (Burkholderiaceae) were ranked the top 10 nodes with high degrees in autumn, but it didn’t occur in the list of top 10 nodes for other three seasons (Additional file 3: Table S3). Planctomycetes (CL500-3 and *Pirellula*) and Kiritimatiellaeota (WCHB1-41) were only included in the top 10 nodes with high degrees in the network of winter, and Verrucomicrobia (Cephaloticoccus) were only ranked in the top 10 nodes with high degrees in the network of spring (Additional file 3: Table S3). Different nodes affiliated to Actinobacteria were included in the top 10 nodes with high degrees in the network of four seasons (3 in winter, 0 in spring, 5 in summer, and 1 in autumn). These specific and distinct microbial taxa played various roles in structuring the ecological networks for each season based on the results at both ASV (Fig. 2) and family (Additional file 9: Fig. S3) levels.

### Microbial co-occurrence in upper Bay versus lower Bay

**Fig. 3:**
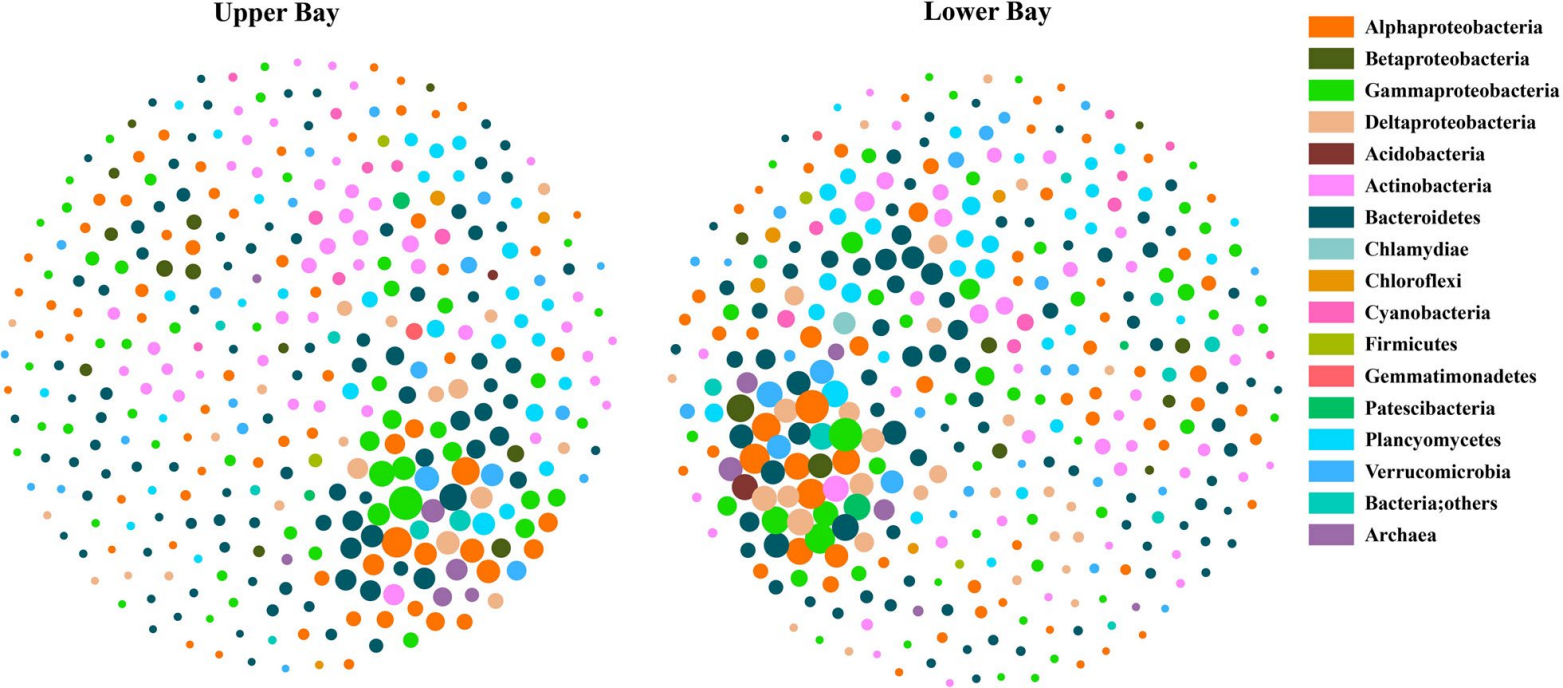
Microbial co-occurrence networks in upper Bay and lower Bay. Color-coded nodes represent major bacterial ASVs. Node sizes indicate number of connections (degree) for each node (ASV). Negative and positive correlations are shown in red or black lines

Similarly for clarity and brevity, only networks based on microbial connections (degree) were shown (Fig. 3). Networks for upper and lower Bay were different regarding size and topology (Fig. 3 and Table 1). The network in lower Bay consisted of 376 nodes and 5, 012 edges (average degree of 26.7), while the network in upper Bay was consisted of 367 nodes and 4, 526 edges (average degree of 24.7) (Table 1). The amount of average path length and graph density means the network of upper Bay was more split compared to the lower Bay (Table 1). Compared to upper Bay, more microbial taxa and more pronounced associations occurred in lower Bay with higher graph density (Table 1).

The top 10 ranked microbial taxa based on degrees and betweeenness ranking were showed in the Additional file 4: Table S4. In upper Bay, the nodes with high-degree were belonged to Gammaproteobacteria (Alcanivorax and OM60(NOR5) clade), Alphaproteobacteria (SAR116 clade, Parvibaculaceae and Sneathiellaceae), Bacteroidetes (Rhodothermaceae), Verrucomicrobia (Pedosphaeraceae), and Deltaproteobacteria (SAR324 cluster) (Additional file 4: Table S4). In contrast, those nodes with high degree in the lower Bay were affiliated with Gammaproteobacteria (Alcanivorax, SAR86 clade and an uncultured Gammaproteobacteria), Alphaproteobacteria (Rhodospirillales, SAR116 clade and Sneathiellaceae and AEGEAN-169 marine group), and Deltaproteobacteria (SAR324 clade(Marine group B)) (Additional file 4: Table S4). Similarly, those nodes with high betweenness centrality were also distinct between upper and lower Bay (Additional file 4: Table S4). Same patterns of microbial networks across space were also observed at the family level. Overall, our results demonstrated that the microbial networks differed between upper Bay and lower Bay, and different microbial taxa contributed to the modularity and stability of the microbiome network structure across space.

### Stability of estuarine microbiome networks

In addition to comparing the topological properties listed in Table 1, we analyzed the responsiveness of network fragmentation (*f*) to removal of top 10 nodes with highest relative abundance, degree and betweenness centrality, respectively. Compared to the original network (*f* = 0.18, number of components = 3), the *f* and the number of components changed more dramatically in the treatment of removing 10 nodes with highest betweenness centrality (*f* = 0.27, number of components = 5) compared to the treatment of removing 10 nodes with the highest relative abundance (*f* = 0.23, number of components = 4) or degree (*f* = 0.19, number of components = 3) (Table 1). Similar results were also observed in modularity (Table 1), suggesting removal of the nodes with high betweenness centrality could much strongly undermine the stability and persistence of the prokaryotic microbial network than removal of the most abundant/highest degree nodes. Further, the degree and betweenness centrality of abundant groups (top 10, 20 and 50, respectively) were significantly different compared to those ASVs with high degree or betweenness centrality (*P* < 0.01), respectively. Combined with the results of Fig. 1, our results clearly showed that the most abundant prokaryotic microbial groups are neither necessarily the hub species nor with high betweenness centrality in CB. Compared to the abundant taxa, the minor/rare groups with high degree or betweenness centrality contribute greatly to the stability of prokaryotic microbial co-occurrence in the Bay.

**Fig. 4:**
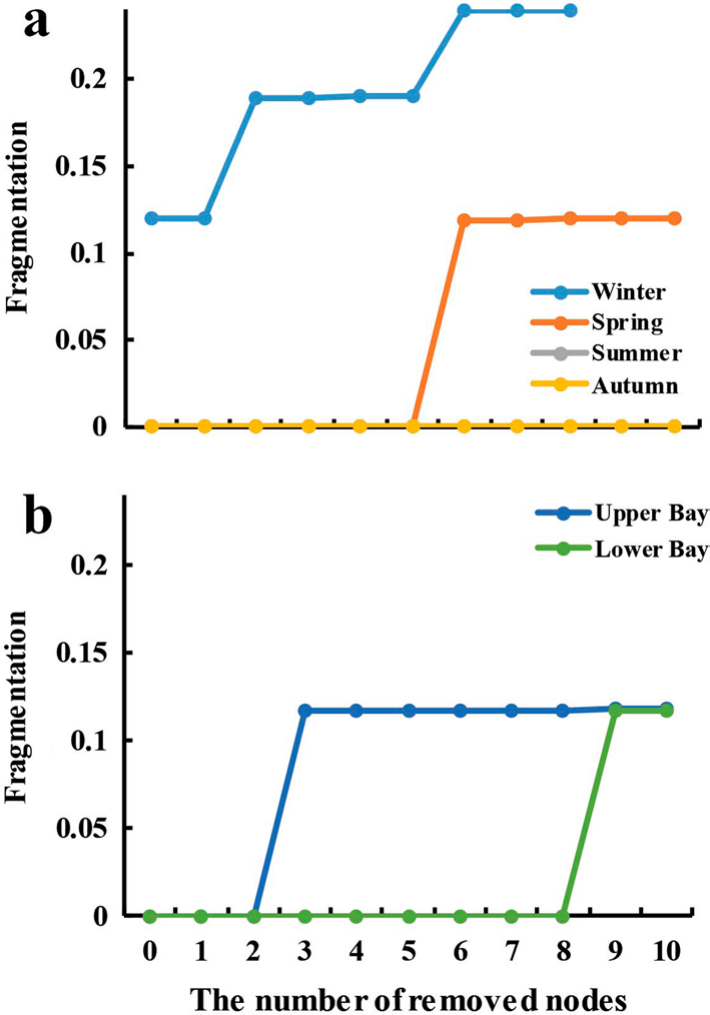
The fragmentations of co-occurrence networks with consecutive removal of 10 nodes with the highest betweenness centrality for four seasons (**a**) and upper/lower Bay (**b**) bacterial networks

We further analyzed the stepwise responsiveness of co-occurrence network fragmentation to removal of the top 10 nodes with highest abundance/degree/betweenness centrality for seasonal and spatial microbial networks. Removing the low abundance taxa (but with high betweenness centrality) greatly reduced the stability of the co-occurrent networks across time and space than the removal of abundant ASVs (*P* < 0.01) (Additional file 5: Table S5). Thus, the contribution of rare taxa to network stability has been further demonstrated in microbial networks across time and space (Fig. 4, and Additional file 5: Table S5). *f* started an increase from the second round in winter, and then *f* increased from the sixth and nineth round. The *f* values in winter were significantly higher compared to those of spring, summer and autumn (*P* < 0.01) (Fig. 4). Similarly, the *f* values in spring were significantly higher compared to those of summer and autumn (*P* < 0.01). Also the number of components increased from 2 to 6 in the network of winter (2 for the spring and 1 for summer and autumn) after the removal processes (Fig. 4 and Additional file 5: Table S5). but it maintained 0 even after 10 removals in summer (Fig. 4 and Additional file 5: Table S5). These results further elaborate that the stability of microbiome networks varied across seasons in the CB, where microbial co-occurrence were more stable and resilient in warm seasons (summer) than those in cold season (especially winter). Similarly, microbial networks showed higher stability in lower Bay than upper Bay (Fig. 4 and Additional file 5: Table S5): the number of components (*f*), and *f* (0.1179) increased greatly in the upper Bay compared to the original network while the network properties in the lower Bay changed little after the removal of top 10 nodes (Additional file 5: Table S5).

**Fig. 5:**
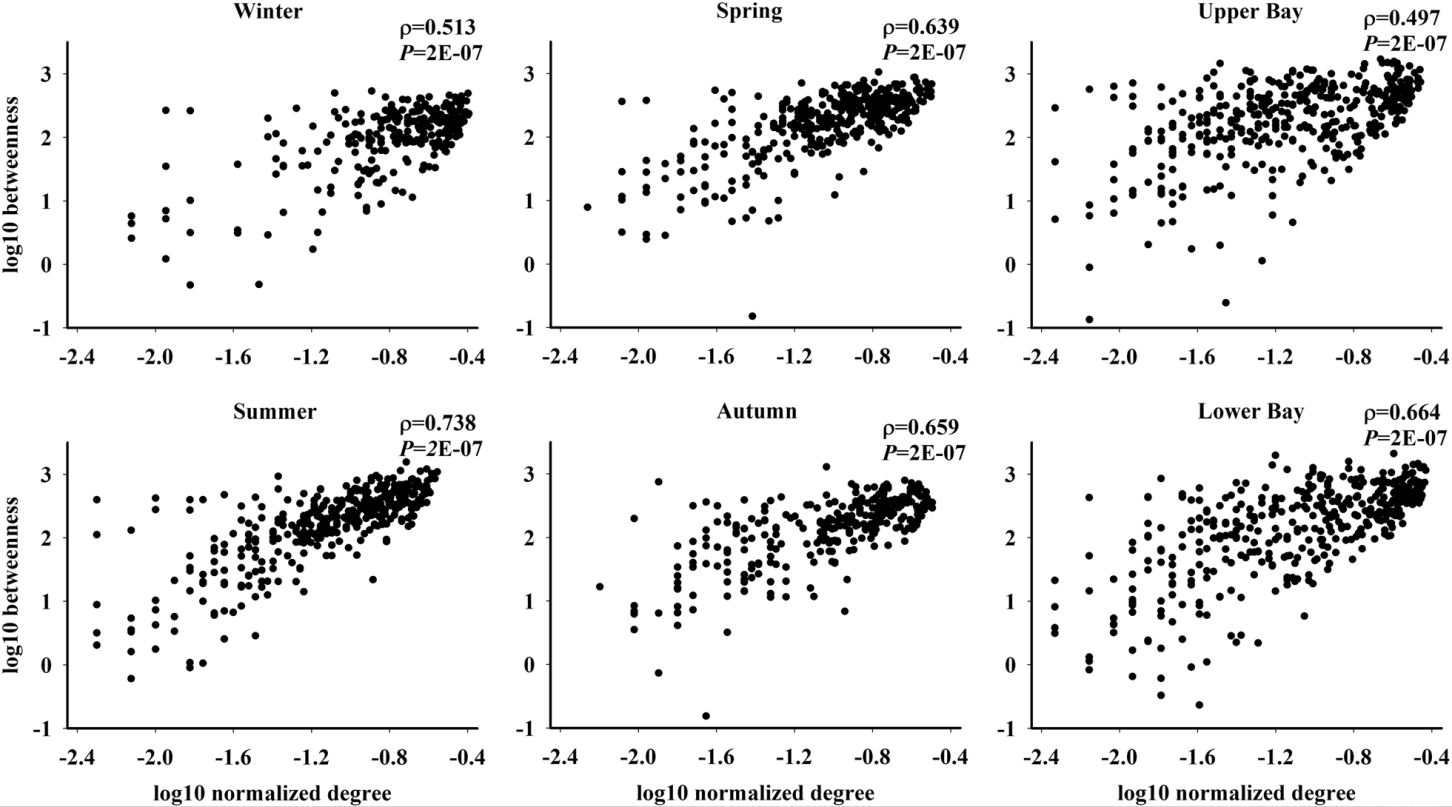
Correlations between normalized degree and betweenness in seasonal and spatial networks

Seasonal networks contained high percentage of significant negative correlations (15.34%-18.65%) than spatial ones (upper Bay, 7.60%; lower Bay, 11.15%) (Table 1). The high proportion of negative correlations in each season could increase the stability and persistence of microbial network. Significant correlations between node-normalized degree and betweenness centrality were observed in each spatial and temporal network based on spearman-correlation analysis (Fig. 5). Further, Monte Carlo simulation results showed that the correlations (ρ values) were significantly different (*P* < 0.01) in microbial networks across time and space: Summer > Autumn > Spring > Winter; and lower Bay > upper Bay. The difference suggested that the nodes with high betweenness centrality (“gatekeepers”) also comprised high degree (*i.e.*, hub species) in warm seasons compared to those in cold seasons, and in lower Bay compared to those in upper Bay. Therefore, once the “gatekeepers” were removed due to disturbances, likely they could be replaced by other hub species, which would connect different compartments and hold the network together. This can help to maintain the stability and enhance the anti-interference ability of the network. In addition, high modularity was observed across seasons (0.404–0.496) compared to spatial scales (0.335–0.363) (*P* = 0.03) (Table 1). Modules could be used to visualize different niches in microbial networks and thus habitat partitioning/preferences strongly existed under different seasons compared to spatial variations in the CB with long residence time.

Overall, the stability of microbial co-occurrence networks were tested and verified through comparisons of microbial networks across spatial and temporal scales. Based on our results of fragmentations, proportion of negative correlations, the correlation between node-normalized degree and betweenness centrality, and modularity, this study showed that the structure, properties, and stability of prokaryotic microbial networks were distinct across spatiotemporal variations. Microbial co-occurrence networks in warm seasons (especially summer) were more stable than cold seasons (*i.e.*, winter), and the lower Bay was more stable than the upper Bay.

### Associations with environmental factors

**Fig. 6:**
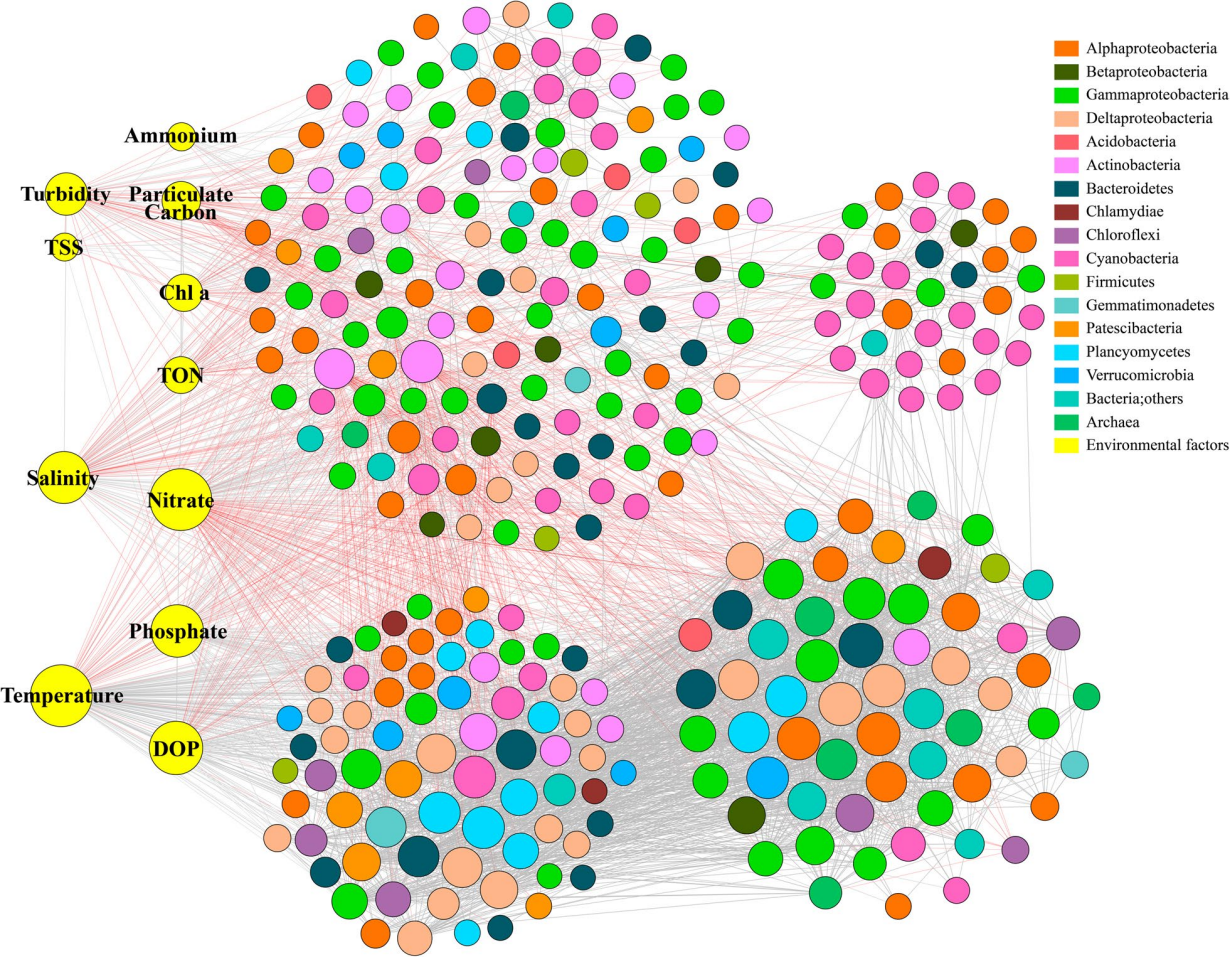
The association network of microbiomes and environmental factors. Color-coded nodes represent major bacterial ASVs. Red lines and black lines indicate significant negative or positive correlations (*P* < 0.05, Q-value < 0.05). Significant and strong correlations (*P* < 0.05, Q-value < 0.05, and |r|≥ 0.6) between microbial taxa are shown. TSS, total suspended solids; TON, total organic nitrogen; DOP, dissolved organic phosphorus

To further explore the effect of environmental variations on microbial networks, microbial network ASVs and their associated environmental factors were included in the further network analysis (Fig. 6). Significant correlations between environmental factors and microbial taxa were visualized by network and clusters (Fig. 6). The majority of taxa (nodes) in the same cluster may have close relationships or share similar ecological niches (*i.e.*, co-occurrence). Seasonal changes (temperature), salinity gradients and TSS (and turbidity) levels were significantly correlated to nutrient availability (nitrogen, phosphorus), PC, and Chl *a* concentrations in the Bay, and all of these factors were responsible for separation and topology of microbial networks across season and space (Fig. 1, 2, 3 and 6). Total 163 taxa were identified closely associated with temperature while 121 strongly responded to salinity. Meanwhile, TSS, nutrient availability (mainly N and P), PC, and Chl *a* also played significant roles in microbial distribution and co-occurrence (Fig. 6).

**Fig. 7:**
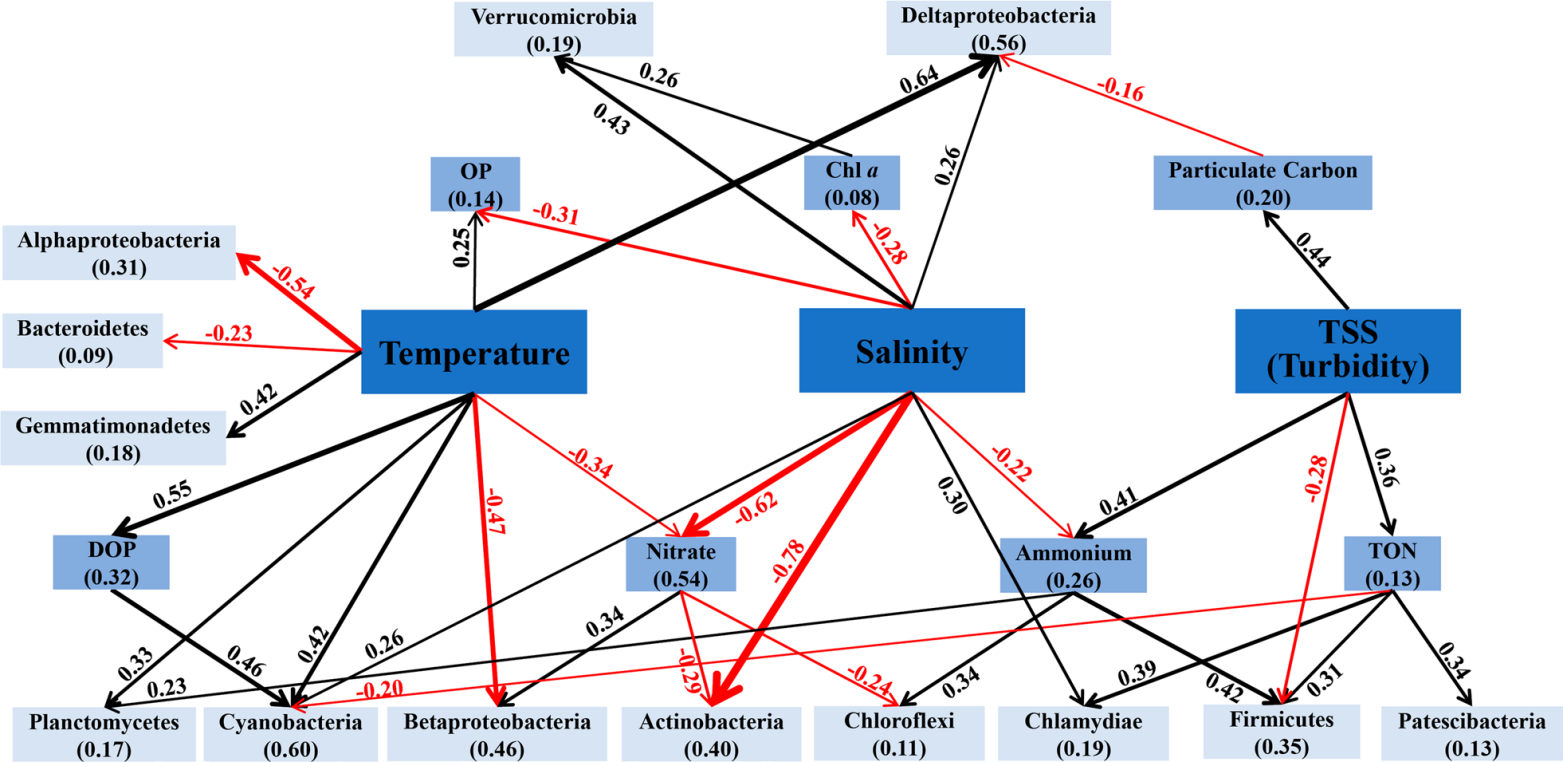
Structural equation model to demonstrate the connections between environmental factors and planktonic microbiome distributions. Red lines, black lines and the associated numbers indicate significant negative or positive correlations and the standard estimates. The thickness of arrows and the number associated with each line represent the strength of correlations. Numbers in parenthesis shows the explanation degree for each factor

MRT results confirmed that temperature, salinity, and TSS were ranked the top drivers to shape spatiotemporal variations of microbiomes in CB (Additional file 11: Fig. S5). In summary, the accumulative variance explained by temperature, TSS and salinity were 39.2%, 12.6%, and 4.9% respectively (Additional file 11: Fig. S5). Based on these results, relationships between microbiomes and major environmental variations were further assessed by piecewise SEM (Fig. 7). Due to the limited space, only key correspondences were highlighted in the Fig. 7, and the others and co-varied relations were included in the supplemental Additional file 6: Table S6. We found that temperature, TSS and salinity contributed greatly to the distribution and associations of microbial groups and also strongly affected other environmental parameters including nutrient availability, PC and Chl *a* (Fig. 7). For instance, increase of temperature was associated with decreased Alphaproteobacteria, Betaproteobacteria and Bacteroidetes (negative effect), and increased abundance of Deltaproteobacteria, Planctomycetes, Gemmatimonadetes and Cyanobacteria (positive effect) (Fig. 7). Through correlations with nitrate and DOP, the temperature also affected other microbial groups such as Cyanobacteria, Actinobacteria and Chloroflexi (Fig. 7). Salinity gradient in the Bay was significantly linked to Actinobacteria, Cyanobacteria, Verrucomicrobia, Deltaproteobacteria and Chlamydiae, while it influenced Chloroflexi, Verrucomicrobia, Firmicutes through reducing the availability of nitrate, ammonium, Chl *a*, and OP (Fig. 7). These direct and indirect effect of environmental variables relative to major bacterioplankton groups in the Bay and the strength of multiple effects were analized by piecewise SEM. Along with PC, ammonium and TON, TSS impacted several groups including Deltaproteobacteria, Chlamydiae, Chloroflexi, Firmicutes and Patescibacteria (Fig. 7). In general, our results clearly showed how spatial and temporal variations of environmental factors affected estuarine microbiome association and co-occurrence (networks) (Additional file 9: Fig. S3, Figs. 6, 7).

## Discussion

The estuarine microbiome networks are comprised of highly interconnected taxa, formed clustered topologies, and thus contained ‘small-world’ properties [77]. Among the interconnected taxa, the abundant microorganisms contribute greatly to the biomass, nutrient cycling, and primary production [11, 78, 79]. However, the most abundant microbial groups are not those taxa that hold the network together, such as “hub species” or “gatekeepers” at both ASV and family levels (Fig. 1 and Additional file 2: Table S2). Thus, abundant taxa may not be necessarily critical to the prokaryotic microbial network structure or their stability (Table 1 and Additional file 2: Table S2). In this study, the hub species and gatekeepers are relatively low in abundance or belong to rare species (Additional file 2: Table S2), but they play fundamental roles in network persistence and contribute greatly to the stability and resilience of these taxa-taxa networks [80]. Recent studies have increasingly emphasized the ecological importance of the rare biosphere, because rare taxa can include more metabolically active microorganisms than abundant taxa (as measured by RNA to DNA ratios), and they may be keystone species in regulating the functioning of aquatic environments [81, 82]. In addition, the rare microbes have been shown to fulfill essential functions associated with nutrient cycling, and may enhance functionality of the abundant microbes [83]. For example, it has been demonstrated that rare species offer the required gene pool to catalyze complex degradation processes of organic compounds, and some pollutants are often degraded by species falling below the detection limit in pristine samples [84].

Our results further corroborated the significance of “hub species” and “gatekeepers” via stability testing with removal processes. The responsiveness of network fragmentation to removal of nodes with highest betweenness centrality provide important insights into the susceptibility of prokaryotic microbial networks to disturbance [46]. Our results suggest that the loss of those potential “gatekeepers” contributes disproportionately to network fragmentation, which essentially agrees with earlier reports on food webs and mutualistic networks showing high fragility/susceptibility upon selective removal of taxa [33, 70, 85]. Sequencing data allowed us identify that potential “gatekeepers” were affiliated with Actinobacteria (Sporichthyaceae, Microbacteriaceae, CL500-29 marine group and PeM15), Betaproteobacteria (Polynucleobacter), Alphaproteobacteria (*Sphingorhabdus*), Bacteroidetes (Cryomorphaceae), Planctomycetes (*Pirellula*), and Gammaproteobacteria (Legionella). These taxa had highest betweenness centrality values (937–1943) and were consistently present in the major component of the co-occurrence networks. The loss of “gatekeepers” may adversely affect the robustness and resistance of microbiome structure and associations [33, 70, 85]. Similarly, strongly interacting species (*i.e.*, “hub species”) are important to CB microbial communities, and they were shown to be able to steer microbiome ecosystems towards certain community types [62]. Additionally, as part of the microbial “seed bank”, rare taxa (including these high degree/betweenness centrality but low abundance species) can potentially drive ecosystem responses to environmental changes and become dominant under favorable conditions [86], therefore providing a mechanism for community persistence and stability [87].

Similar to microbiome structures [14, 54], microbial co-occurrence networks showed strong temporal and spatial patterns with distinct property and stability (Fig. 2–5, Additional file 8-Additional file 10: Fig S2–S4 and Table 1). Our results showed that consistent patterns of microbial networks were observed at different taxonomic levels. These differences are likely due to strong gradients in seasonality, salinity, nutrient availability, and other causal environmental factors [11, 18, 88, 89]. CB, the largest estuary in northeast America, experience typical seasonal changes and constant freshwater/oceanic water input and exchange. The dynamic estuarine gradients lead to large variations in bulk bacterial production and biomass [19, 20], and community composition [11, 22], and subsequently affecting the property and stability of microbial networks (this study, Fig. 2–6 and Table 1; [52, 90, 91]).

Warm season microbial networks revealed high number of edges, high average degree, hign graph density, low number of components and clusters, low fragmentation, high modularity, and high number of negative links. Stronger correlations between node-normalized degree and betweenness centrality and less variability of fragmentation after removing the potential 10 “gatekeepers” were also observed in the networks for summer and autumn (Fig. 4 and Table 1). These results indicated that microbial networks were more stable during warm seasons compared to those in winter and spring. Microbial co-occurrence affected by temporal variations were also found in Alaska Beaufort Sea coast [51], the San Pedro Ocean Time-series station [90], and the Lake Mendota [92]. The higher number of nodes in the co-occurrence networks of warm season agrees with the previously reported high microbial diversity in the Bay [14, 54]. High biodiversity is able to promote co-occurrence between microbial communities [93], and these biotic associations including competition, are commonly thought to increase co-occurrence in microbial networks as they refer to common resources and environmental conditions [30, 94, 95]. In addition, increase in grazer richness has a positive effect on the bacterial richness and evenness [96], stemming primarily from the widespread distribution of resources and then resulting in ecological niche complementarity [97–99]. Finally, this stability is also due to long residence time and relatively high growth rate (and mortality) of different microbial populations [20, 89], which allows estuarine bacteria overwhelm those allochthonous populations from marine or freshwater [100]. Therefore, stable prokaryotic microbial networks in warm season in the Bay may arise from a better balance of the microbial associations (*e.g.*, mutual, competitive and prey) and nutrient availability during summer and autumn than winter and spring [11, 20, 101]. In contrast, microbial associations in cold seasons contained high fragmentation and it may be reinforced by stronger nutrient limitations and lower growth rates (due to low temperature) [20].

Compared to the upper Bay, the network in the lower Bay was more connected with more negative correlations (Fig. 3, Table 1). High percentage of negative links in the networks could stabilize co-oscillation within microbial communities and increase network stability [31]. A strong association between node-normalized degree and betweenness centrality and the less variability of fragmentation after removing potential “gatekeeper” nodes were also observed in the network of lower Bay (Fig. 4, 5). Our results suggested that the stability of prokaryotic microbial network was higher in the lower Bay compared to the upper Bay. The stability difference between upper and lower Bay could be due to the disturbance and interference from freshwater vs. oceanic water. Similar environmental variations were also found in many other estuaries, and spatial variation also could affect bacterial associations, including Ems estuary [91], Hangzhou Bay [43, 53] and Pearl River Estuary [103]. River input, land runoff, suspended particles/turbidity, and accompanied nutrients availability are all pulsating with strong seasonal/inter-annual variations and uncertain patterns in the upper Bay. Conversely, salty water intrusion from the North Atlantic Ocean can be relatively consistent, and we thus hypothesize that less temporal disturbances from the ocean contribute to higher stability of microbial networks in lower Bay compared to the upper Bay.

Estuarine microbiomes continuously experience environmental perturbations including pulse, press and environmental stochasticity [104]. This study was not originally designed and intended to investigate community recovery after defined perturbations such as pulse (e.g., floods) or press (lasting disturbances such as climate changes), therefore the results are more reflecting the ecological stability of microbiome correlations under the perturbation of environmental stochasticity [104]. Ecological stability is a multidimensional concept that captures different aspects of the dynamics of the ecosystem and its response to perturbations [105]. Pimm (1984) summarized five components of ecological stability that are in common use: asymptotic stability, variability, persistence, resistance, and resilience [106]. In this study, multiple metrics in network topology including the number of components, modularity, negative correlations, fragmentation as well as the correspondence between node-normalized degree and betweenness centrality are used to characterize the asymptotic stability and variability, both of which indicate higher network stability in warm season than cold seasons, and lower Bay than upper Bay. However, due to the high correlations between metrics used, it is difficult to propose a single best way of selecting metric(s) in evaluating the independent stability components. For instance, negative correlations between metrics would suggest trade-offs, although they seem to be rare exceptions in complex trophic communities [105]. Further, the choice of the metrics always depends on the system studied and practical constraints, therefore measuring more stability metrics with deep and hierarchical analyses and/or explained variance analyses can help making informed choices and improve our understanding of the full-picture of microbial stability in the Bay for the future studies.

Due to the continuous environmental perturbations, the estuarine microbiomes and their distribution are a comprehensive output of the bio-associations between microbial populations and the response to the surrounding environmental gradients. Our results show that shifts of environmental factors (including temperature, salinity, TSS, nitrogen, phosphorus, and PC) have strong effect on microbial community structure and networks. The shifts within the spatiotemporal variations seem to favor strong co-occurrence patterns (lower fragmentation and higher stability) of prokaryotic microbial associations, implying elevated biotic co-occurrence or species sorting strongly mediated by the local environment [95, 108]. Physiologic predisposition and nutritional tolerance of microbiomes tend to maintain stable communities inter-annually during certain seasons [11, 25, 109]. Focusing on the identified prokaryotic microbial networks and their responses to environmental variations could provide us valuable insights into the microbiome adaptation and habitat partitioning/preferences in the Bay across seasonal changes and spatial gradients [30, 67, 110]. In addition, co-occurrence networks reveal critical information on co-oscillation between microbial taxa and also the stability of these involved communities in the Bay [30, 36, 110]. Therefore, changes in estuarine microbial networks resulting from disturbances provide a potential to examine the legacy effect on estuarine microbiome population structure, ecological function (*e.g.*, contribution to primary productivity, food webs and economical sustainability) and its vulnerability to future disturbances, such as anthropogenic influence (*e.g.*, eutrophication, contamination and damming) and climate change (*e.g.*, drought and flood) [11, 31, 110].

To the best of our knowledge, this is the first systematic and thorough study of the microbiome co-occurrence associations, their stability, and the corresponding environmental factors in an estuary with long residence time, the Chesapeake Bay. There are some limiations and caveates in this study. For instance, most environmental parameters in the current study were not from the on-site samples that collected during the cruises. Instead, we used the data from the long term Chesapeake Bay Program’s (CBP) Water Quality Database (https://www.chesapeakebay.net/what/downloads/cbp_water_quality_database_1984_present). Plenty of previous studies and our own observations have shown clear and repeatable spatiotemporal patterns in the Bay [111–113]. These stable patterns are reoccurring annually and spatially, and therefore the CBP data closest to our sampling sites and dates were used in our analysis. In addition to the environmental gradients included in this study, many other abiotic or biotic associations also play critical roles in microbiome composition and distribution. For example, grazing and viral lysis are important factors that may affect the associations and stability of estuarine microorganisms [114–116], which deserve future investigations. Currently, we are analyzing the structure of microeukaryotic communities in the Bay, which may provide further information on primary producers and their interactions with other estuarine microbiomes. All in all, examining the natural spatiotemporal changes and stability of microbial networks as well as their interactions with other organisms is essential to strengthen the understanding of estuarine ecosystem processes.

## Conclusions

This study shows that environmental variations dictate stable and resilient estuarine microbiome associations in the Chesapeake Bay. Compared to dominant taxa, rare groups (*e.g.*, hub species and gatekeepers) contribute greatly to the network topology and stability. Microbiomes in warm seasons exhibit stronger and more interactive networks than cold seasons, while the microbial network in the lower Bay is more stable than the upper Bay. The freshwater input from upstream brings pulsating and uncertain factors to microbial populations in CB, such as nutrients, particles, and microorganisms from terrestrial and freshwater environments. In contrast, the intrusion of seawater at the lower Bay is relatively mild and consistent so that the Bay microbiomes are able to adapt to the subsequent impact. Our piecewise structure equation model (SEM) unraveled that spatiotemporal variations (*i.e.*, temperature, salinity, TSS, nutrient availability) are the primary driving factors for microbiome structures and co-occurrence, and these environmental gradients manipulate the stability and tolerance/susceptibility of estuarine microbiota. This work shows, for the first time, dynamics of microbiome networks and their responses to environmental gradients in a typical estuarine environment with long residence time. Given such dynamic environmental gradients, stable and persistent microbiome networks suggest that biotic associations play a central role in maintaining integrity and resilience of estuarine microbiomes.

## Supplementary Information

**Additional file 1: Table S1.** Description of the stability metrics used in this study.

**Additional file 2: Table S2.** Top 10 nodes based on abundance/degree/betweenness centrality in the network of all samples

**Additional file 3: Table S3.** Top 10 nodes with highest abundance/degree/betweenness centrality in each season

**Additional file 4: Table S4.** Top 10 nodes with the highest abundance/degree/betweenness centrality in upper Bay and Lower Bay

**Additional file 5: Table S5.** Stepwise changes of network properties with removal of top ranked 10 nodes (betweenness centrality)

**Additional file 6: Table S6.** Correspondence of microbial groups and environmental factors for structural equation model

**Additional file 7: Fig. S1.** Map of the Chesapeake Bay showing sampling stations.

**Additional file 8: Fig. S2.** Co-occurrence networks of planktonic microbiomes in the Chesapeake Bay. Color coded nodes represent major bacterial families. The relative abundance, degree and betweenness centrality of each family are shown by node sizes.

**Additional file 9: Fig. S3.** The architecture of microbial networks in winter, spring, summer, and autumn. Color-coded nodes represent major bacterial families. Node sizes indicate number of connections (degree) for each node (family).

**Additional file: 10 Fig. S4.** Microbial co-occurrence networks in upper Bay and lower Bay. Color-coded nodes represent major bacterial families. Node sizes indicate number of connections (degree) for each node (family).

**Additional file 11: Fig. S5.** The multivariate regression tree (MRT) for Chesapeake Bay microbial samples with explanatory variables (environmental data). Two red numbers indicate the split times, and the variance explained by each split. The black numbers show the average frequency of all taxa in the samples and the following “n” number is the total sample number before the split.

## Data Availability

All data generated or analyzed during this study are included in this published article and its supplementary information files. The high throughput sequencing data for the Chesapeake Bay microbiomes are available through the GenBank data database under the Accession Numbers SRX6973110-SRX6973185.

## Abbreviations

CB: Chesapeake Bay
MRT: Multivariate regression tree
SEM: Structural equation model
DNA: Deoxyribonucleic acid
CTD: Conductivity temperature depth
CBP: Chesapeake Bay Program
TON: Total organic nitrogen
Chl *a*: Chlorophyll *a*
DOP: Dissolved organic phosphorus
OP: Orthophosphate phosphorus
TSS: Total suspended solids
PC: Particulate carbon
rRNA: Ribosomal ribonucleic acid
Qiime: Quantitative insights Into Microbial Ecology
ASVs: Amplicon sequence variants
*f*: Fragmentation
CL: Disconnected subgraphs
*N*: Overall number of nodes
ANOSIM: Analysis of similarities
