# Supplementary figures and images for: Estuarine gradients dictate spatiotemporal variations of microbiome networks in the Chesapeake Bay
Source: Environ Microbiome. 2021 Nov 27;16:22. doi: 10.1186/s40793-021-00392-z (PMC8627074; doi:10.1186/s40793-021-00392-z)

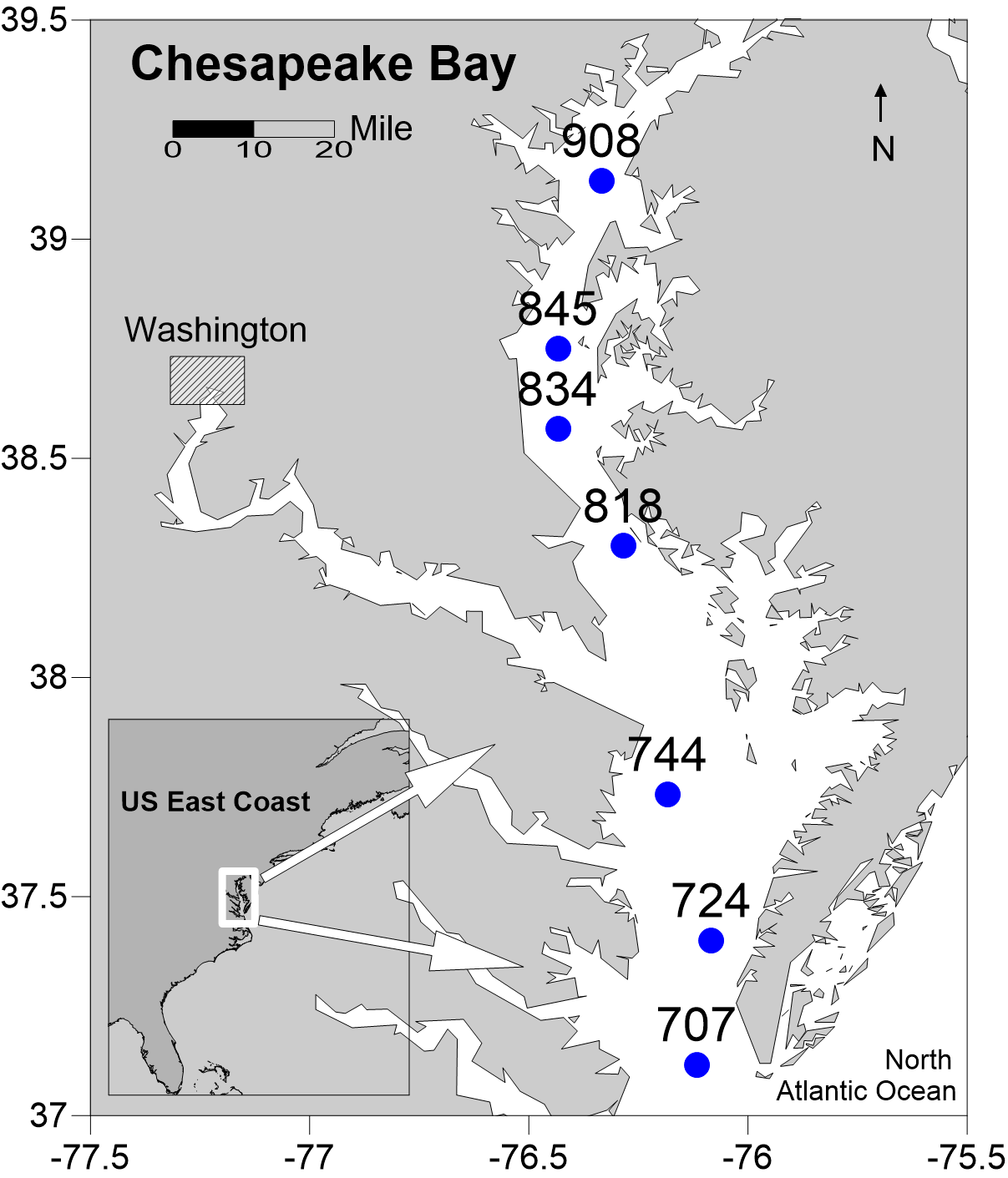

Supplement: Supplementary file 7 — Additional file 7: Fig. S1. Map of the Chesapeake Bay showing sampling stations. [file 40793_2021_392_MOESM7_ESM.tiff]

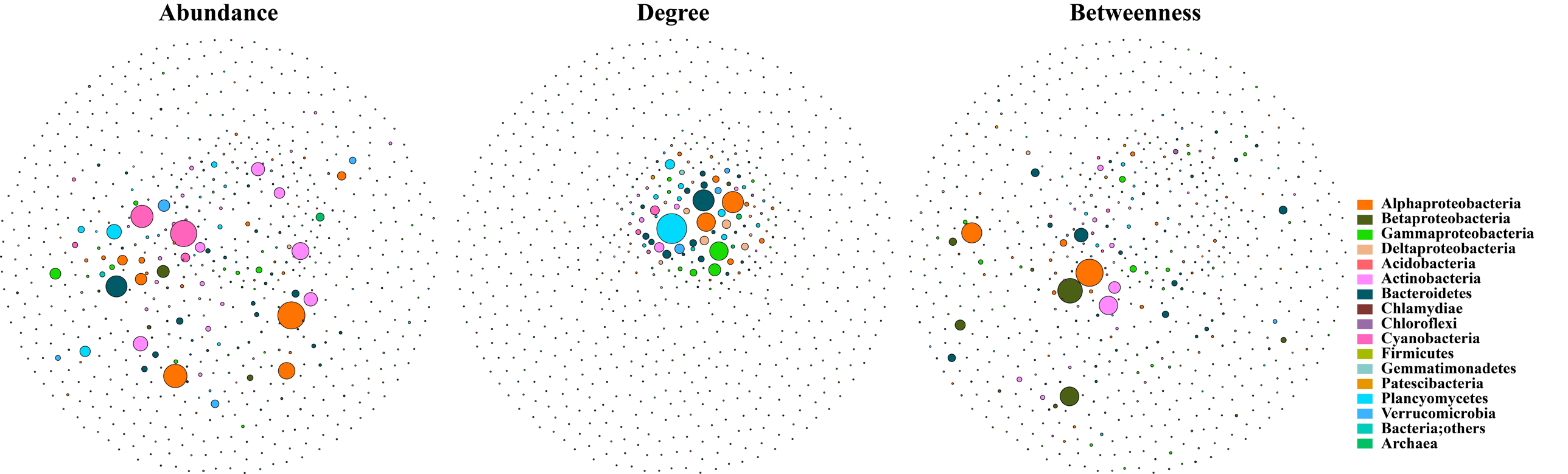

Supplement: Supplementary file 8 — Additional file 8: Fig. S2. Co-occurrence networks of planktonic microbiomes in the Chesapeake Bay. Color coded nodes represent major bacterial families. The relative abundance, degree and betweenness centrality of each family are shown by node sizes. [file 40793_2021_392_MOESM8_ESM.jpeg]

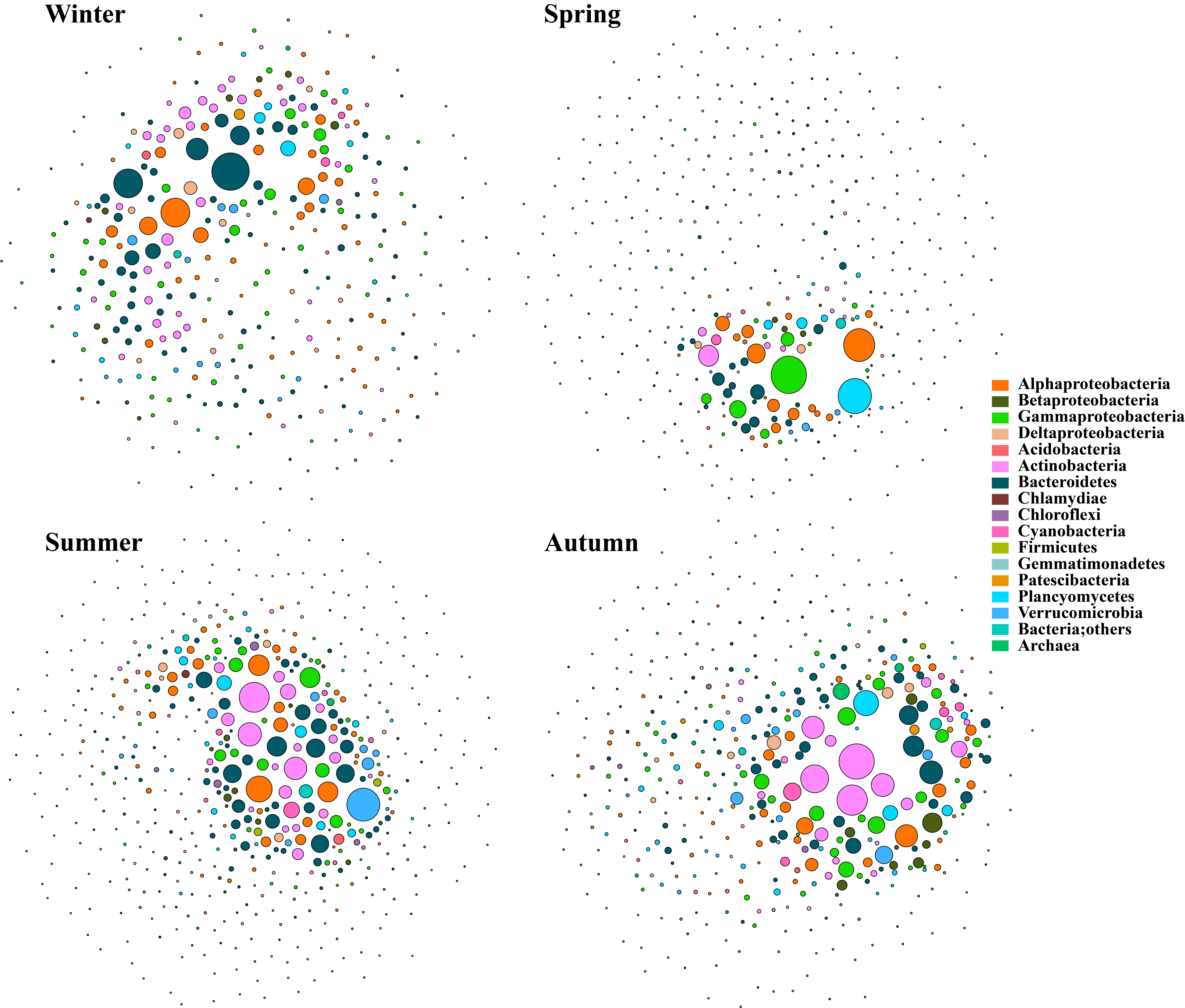

Supplement: Supplementary file 9 — Additional file 9: Fig. S3. The architecture of microbial networks in winter, spring, summer, and autumn. Color-coded nodes represent major bacterial families. Node sizes indicate number of connections (degree) for each node (family). [file 40793_2021_392_MOESM9_ESM.png]

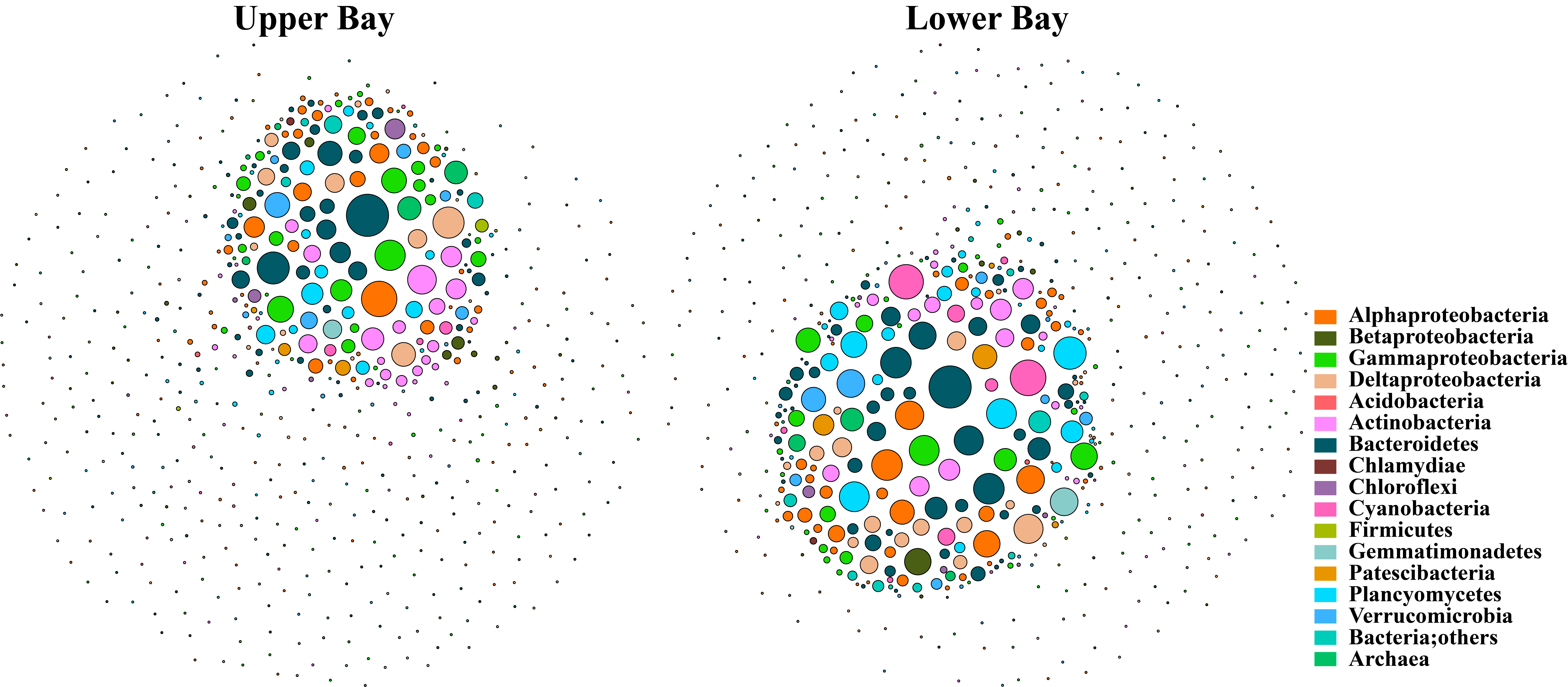

Supplement: Supplementary file 10 — Additional file: 10 Fig. S4. Microbial co-occurrence networks in upper Bay and lower Bay. Color-coded nodes represent major bacterial families. Node sizes indicate number of connections (degree) for each node (family). [file 40793_2021_392_MOESM10_ESM.png]

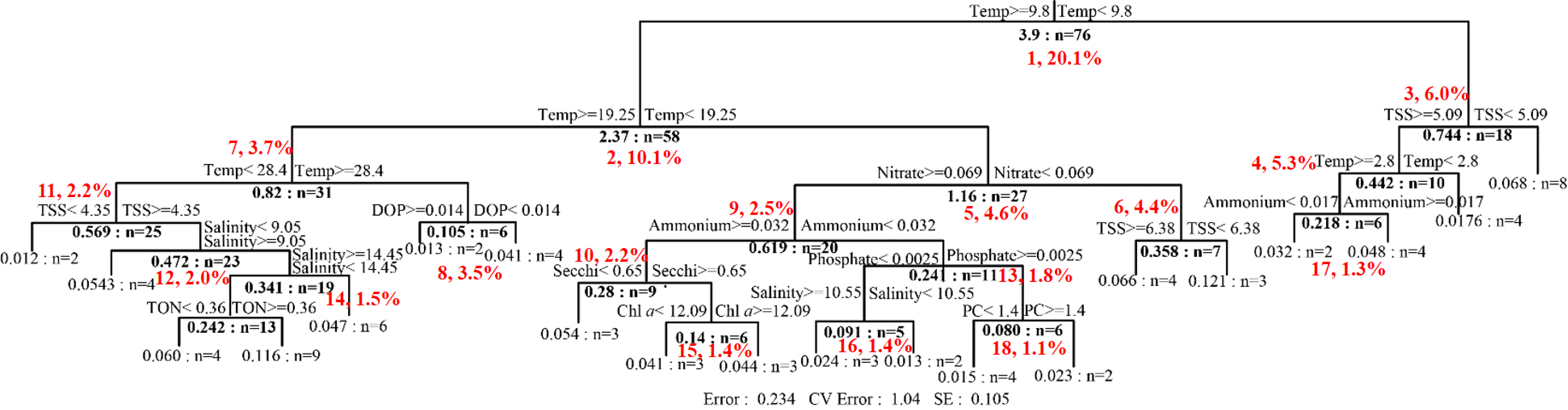

Supplement: Supplementary file 11 — Additional file 11: Fig. S5. The multivariate regression tree (MRT) for Chesapeake Bay microbial samples with explanatory variables (environmental data). Two red numbers indicate the split times, and the variance explained by each split. The black numbers show the average frequency of all taxa in the samples and the following “n” number is the total sample number before the split. [file 40793_2021_392_MOESM11_ESM.tiff]
